# A systematic review of gait perturbation paradigms for improving reactive stepping responses and falls risk among healthy older adults

**DOI:** 10.1186/s11556-017-0173-7

**Published:** 2017-03-02

**Authors:** Christopher McCrum, Marissa H. G. Gerards, Kiros Karamanidis, Wiebren Zijlstra, Kenneth Meijer

**Affiliations:** 1 NUTRIM School of Nutrition and Translational Research in Metabolism, Maastricht University Medical Centre+, Department of Human Movement Science, Maastricht, The Netherlands; 2 Institute of Movement and Sport Gerontology, German Sport University Cologne, Cologne, Germany; 3 azM Herstelzorg Centre for Geriatric Rehabilitation and Care, Maastricht, The Netherlands; 4 Sport and Exercise Science Research Centre, School of Applied Sciences, London South Bank University, London, UK

**Keywords:** Adaptation, Aged, Ageing, Biomechanics, Falls, Locomotion, Motor learning, Postural balance, Rehabilitation, Systematic review

## Abstract

**Background:**

Falls are a leading cause of injury among older adults and most often occur during walking. While strength and balance training moderately improve falls risk, training reactive recovery responses following sudden perturbations during walking may be more task-specific for falls prevention. The aim of this review was to determine the variety, characteristics and effectiveness of gait perturbation paradigms that have been used for improving reactive recovery responses during walking and reducing falls among healthy older adults.

**Methods:**

A systematic search was conducted in PubMed, Web of Science, MEDLINE and CINAHL databases in December 2015, repeated in May 2016, using sets of terms relating to gait, perturbations, adaptation and training, and ageing. Inclusion criteria: studies were conducted with healthy participants of 60 years or older; repeated, unpredictable, mechanical perturbations were applied during walking; and reactive recovery responses to gait perturbations or the incidence of laboratory or daily life falls were recorded. Results were narratively synthesised. The risk of bias for each study (PEDro Scale) and the levels of evidence for each perturbation type were determined.

**Results:**

In the nine studies that met the inclusion criteria, moveable floor platforms, ground surface compliance changes, or treadmill belt accelerations or decelerations were used to perturb the gait of older adults. Eight studies used a single session of perturbations, with two studies using multiple sessions. Eight of the studies reported improvement in the reactive recovery response to the perturbations. Four studies reported a reduction in the percentage of laboratory falls from the pre- to post-perturbation experience measurement and two studies reported a reduction in daily life falls. As well as the range of perturbation types, the magnitude and frequency of the perturbations varied between the studies.

**Conclusions:**

To date, a range of perturbation paradigms have been used successfully to perturb older adults’ gait and stimulate reactive response adaptations. Variation also exists in the number and magnitudes of applied perturbations. Future research should examine the effects of perturbation type, magnitude and number on the extent and retention of the reactive recovery response adaptations, as well as on falls, over longer time periods among older adults.

**Electronic supplementary material:**

The online version of this article (doi:10.1186/s11556-017-0173-7) contains supplementary material, which is available to authorized users.

## Background

Falls are a leading cause of injury among older adults, with hip fractures [1–4] and head injuries [1, 3] among the more severe consequences. Falls most often occur during walking [5–9], which is also the most common activity prior to falls that lead to injury or hospital admission [1, 2]. Slipping and tripping during walking are the most common causes of falls among older adults [4, 5, 7, 9–11], which represent failures to predictively (before the perturbation) or reactively (after the perturbation) adapt to changes and challenges in the environment. Therefore, there is a need to physically prepare older adults for situations where unexpected mechanical disturbances to gait could occur.

Lower limb muscle strength [12–21] and tendon stiffness [18] have been associated with stability recovery performance following different balance and gait perturbations, with greater muscle strength and tendon stiffness generally showing modest associations with more effective stability maintenance or recovery. General exercise interventions (combinations of strength, balance and aerobic exercises) have generally resulted in moderate reductions (14–17%) in falls incidence among older adults [22–24], which reflects the modest associations observed between muscle-tendon properties and stability performance. While these reductions are statistically significant, the fact that greater reductions in falls incidence are not seen may be explained by the fact that general exercise interventions often lack specificity to the balance recovery mechanisms that are needed following balance loss, such as compensatory stepping, counter rotation or grasping actions [25, 26]. Balance maintenance requires a complex interaction of several mechanisms and hence, improved balance control in one task in particular is not likely to be of benefit during other tasks [27]. Accordingly, only negligible associations between static posturography and dynamic stability performance (forward lean-and-release and slip/trip recovery tasks) have been reported [28–31]. Therefore, testing and training tasks more specific to balance recovery mechanisms may provide more insight and benefit for falls reduction and prevention.

It has previously been suggested that training involuntary compensatory recovery responses following sudden perturbations is more task specific than general strength and balance exercise for preventing a fall after a loss of balance [32–35]. Even voluntary stepping exercise, such as multidirectional stepping to targets, is not as specific as involuntary, reactive compensatory stepping where faster movement speeds and an inability to make use of anticipatory postural adjustments are characteristic [34–36]. Eliciting involuntary, reactive compensatory stepping by applying unexpected perturbations during walking also increases the task specificity, as most falls occur during walking [5–9]. However, in order to benefit from this kind of training, the participants must be capable of adapting their reactive recovery responses during gait.

Reactive recovery responses are required to cope with unexpected perturbations to gait in order to continue safe locomotion. We define reactive recovery responses here as feedback-driven adaptations in gait in response to mechanical disturbances to the regular gait pattern. The first step of such a response is recognising the onset of the perturbation, achieved though integration of visual, somatosensory and vestibular sensory information. The contribution of each sensory system may vary with perturbation type due to differences in the perception of motion [37]. Stability can then be recovered through a number of strategies, such as compensatory stepping, counter rotation or grasping actions [25, 26]. In situations where compensatory stepping is required to maintain balance, the spatiotemporal characteristics of the step (e.g. direction, timing and amplitude) need to match the requirements for optimal control of stability given the specific environmental constraints. Such reactive responses appear to involve spinal locomotor networks, as chronic spinal cats [38, 39], as well as human infants prior to independent walking [40] exhibit well organised reflex responses (increased swing limb flexion and limb flexor activation) to paw and foot touches during leg swing that simulate a potential trip hazard. Critically, adaptation of these responses has been reported following repeated paw and foot (dorsum) touches in spinal cats [41] and human infants [42], suggesting that spinal locomotor networks are plastic. Therefore, older adults’ reactive recovery responses following gait perturbations may, in part, be improved with repetition via these reflexes. While there is evidence to suggest that certain neurological patient populations may be limited in their reactive adaptation potential during gait (patients with vestibulopathy [43] and Parkinson’s disease [44], for example), the ability to adapt in a reactive or predictive manner to repeated perturbations appears to be largely unaffected by non-pathological ageing [43, 45–50].

When applied in prevention and rehabilitation settings, the use of sudden, unexpected mechanical perturbations during stance or gait is often termed perturbation-based balance training [34, 51–53]. The goal of such training is to target the specific mechanisms of balance recovery related to reducing falls such as compensatory stepping, counter rotation or grasping actions [25, 26]. Aside from these movement strategies to maintain balance, factors such as reaction time, perception of losses of balance and speed of sensory information processing are challenged and may improve with perturbation training. Two recent meta-analyses of randomised controlled trials (RCTs) have reported significantly lower post-training falls incidence among older adults who took part in such training (note that Okubo et al. [54] also included voluntary stepping training interventions in their meta-analysis) [53, 54]. These meta-analyses focussed on RCTs that assessed falls incidence, which may mean that non-RCT studies or studies that did not report falls data, but nonetheless included relevant information on reactive recovery responses following perturbations, may have been omitted. Additionally, of the included studies in these meta-analyses, only four were conducted with healthy older adults and applied sudden, unexpected perturbations during walking [55–58], three of which used very similar perturbation paradigms. As a result, it is difficult to determine, based on these studies, the variety, characteristics (e.g. perturbation type, magnitude, standardisation, scaling, progression etc.) and effectiveness of gait perturbation paradigms that could be used with older adults for improving reactive recovery responses and preventing or reducing falls. Therefore, we systematically searched for all studies that applied unexpected mechanical disturbances during walking in healthy older adults and assessed changes in reactive recovery responses or falls incidence, in order to determine the variety, characteristics and effectiveness of methods that have been used to date for improving reactive recovery responses during walking (using spatiotemporal or biomechanical parameters) and reducing falls (defined using the number of daily life or laboratory-induced falls after exposure to the perturbation paradigms) among healthy older adults.

## Methods

**Fig. 1:**
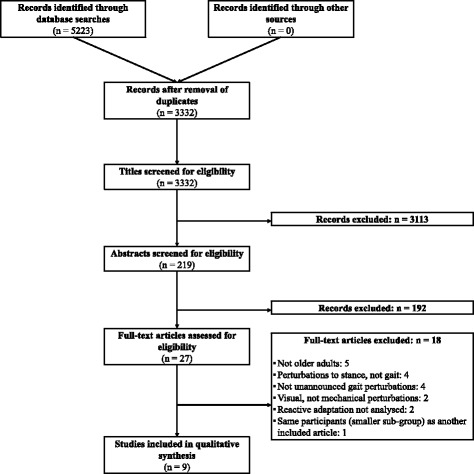
Flowchart of systematic search and article inclusion and exclusion process

A systematic search of PubMed, Web of Science, MEDLINE and CINAHL databases was conducted with sets of terms relating to gait (gait, locomotion, walk, walking), perturbations (agility, balance loss, dynamic balance, dynamic stability, perturb*, slip*, surface translation, trip, tripping, waist pull), ageing (age, ageing, aged, aging, elderly, old, older, senior), and adaptation or training (adaptation, adaptive, adjustments, exercise, rehabilitation, repeated, repetition, responses, task, training). An additional file detailing the search terms for each database is available (Additional file 1). This broad range of terms was used due to the large variance in terminology used in the literature to describe the tasks and underlying mechanisms of interest, as well as the fact that reactive stability tasks are not always specifically described in the titles and abstracts of larger intervention studies. The initial search was conducted on December 16th 2015, with the final check for recent literature conducted on May 18th 2016. Two of the authors independently screened titles, abstracts and full texts for inclusion. It was planned that disagreements regarding inclusion would be discussed and when an agreement could not be reached, a third author would be consulted, but this was not required as the two authors agreed on the articles included and excluded. Inclusion criteria stipulated that the studies were conducted with healthy participants with a mean age of 60 years or older, that the studies applied repeated mechanical perturbations of an unpredictable or unannounced nature during walking, and that reactive recovery responses to gait perturbations or the incidence of laboratory or daily life falls were recorded. The inclusion process for this review, including the number of articles excluded at each stage can be seen in Fig. 1. Once the articles to be included were finalised, a risk of bias assessment using the Physiotherapy Evidence Database (PEDro) Scale [59, 60] was carried out for each article. The PEDro website was consulted and when scores for the included articles were available, these were used. For the remaining articles, two authors independently scored the articles and then compared and discussed the scores before finalising them. Following this, the level of evidence was determined (as described by Teasell et al. [61]) for each type of perturbation used in the included studies.

## Results

### Systematic search results

The complete search and inclusion process can be seen in Fig. 1. The search yielded 5223 records, which was reduced to 3332 after duplicates were removed. The title screening excluded 3113 records, after which the remaining 219 articles’ abstracts were assessed for inclusion. 27 full texts were then assessed and nine articles met all inclusion criteria. The reasons for exclusion at the full text screening stage can be found in Fig. 1.

### Summary of included studies

The systematic search and inclusion process yielded nine studies that met all inclusion criteria. A summary of the participants, the perturbation paradigms, the assessment methods for reactive recovery responses and falls incidence, and the main results of the included articles are reported in Table 1. In these nine studies, moveable floor platforms [55, 57, 58, 62, 63], ground surface compliance changes [64, 65] and treadmill belt accelerations or decelerations [56, 66] were used to perturb the gait of older adults. Eight of the studies used a single session of perturbations [55, 57, 58, 62–66], with two studies using multiple sessions [55, 56]. Eight of the studies reported improvement in some measure of the reactive recovery response to the perturbations [55, 56, 58, 62–66]. Four studies observed a reduction in the percentage of laboratory falls from the pre- to post-perturbation experience measurements [55, 58, 62, 63] and two studies reported a reduction of daily life falls [56, 57]. While most of the included studies were conducted with healthy, community dwelling older adults, it is important to note that Lurie et al. [56] included participants referred for gait and balance training by their primary care provider, but no specific diagnoses or conditions were mentioned.

**Table 1:** Summary of the perturbation paradigms and results of the included studies

Study	Participants Exposed to Perturbations	Perturbation Paradigm		Reactive Response Assessment and/or Falls Monitoring	Main Results
		Perturbation Type/Magnitude	Protocol		
Bhatt et al. [55]	Single session: *n* = 25, 13♀, 73.42 ± 5.42y. Dual session: *n* = 13, 7♀, 70.13 ± 4.75y. All community dwelling, healthy older adults	Slip (low friction moveable platform; slid up to 90 cm forward at foot contact).	Single session: 24 slips in 37 gait trials. Dual session: as above, plus 1 slip trial 3mo later.	Stability, loss of balance & hip height during slip 1 & 24, & the single slip at +6mo. % of falls following the lab perturbations.	Improvement in the observed parameters from slip 1 to 24. Retention in all parameters at +6mo, greater for the dual group.
Bierbaum et al. [64]	13♂, 67.4 ± 3.4y. Community dwelling, healthy.	Surface change perturbation (17 cm thick foam with an average of 10 cm deformation).	19 trials with the 2nd, 8th & 19th as a hard surface, & the rest soft surface.	MoS, BoS & X_CoM_ at touchdown of perturbed & recovery steps.	Improvement across trials in the outcome parameters.
Bierbaum et al. [65]	14♂, 67.3 ± 4.2y. Community dwelling, healthy.	Surface change perturbation (17 cm thick foam with an average of 10 cm deformation).	28 trials: 23 with hard surface, 5 with soft surface.	MoS, BoS & X_CoM_ at touchdown of the perturbed & recovery steps.	Improved MoS of the recovery step for the 4th & 5th perturbations compared to the 1st.
Lurie et al. [56]	*n* = 26, 13♀, 81 ± 6.53y. Healthy older adults referred for gait and balance training.	Anterior/posterior treadmill accelerations of progressive magnitude (scale of 1–5, exact values not reported).	5.84 sessions of 44.25mins (means). Therapist determined perturbation type (stance or gait), magnitude & number.	Mean perturbation magnitude successfully negotiated per session. Retrospective falls data 3mo preceding & for 3mo after the intervention.	Improved mean trip magnitude from 2.44 to 3.44. Non-significant difference in subjects experiencing falls (19% vs. 33%) compared to controls.
Pai et al. [62]	*n* = 38, 19♀, 71 ± 5y. Community dwelling, healthy.	Slip (low friction moveable platform; slid up to 90 cm forward at foot contact).	24 slips in 37 gait trials.	Stability, loss of balance & hip height 300 ms after perturbation onset. % of falls following the lab perturbations.	Reduction in falls & backward losses of balance across trials. Improvement in limb support & stability in the first 3 trials with no further improvement.
Pai et al. [57]	*n* = 67, 44♀, 72 ± 5.5y. Community dwelling, healthy.	Slip (low friction moveable platform; slid up to 90 cm forward at foot contact).	24 slips in 37 gait trials.	Retrospective falls data 12mo preceding & 12mo prospective following the session.	Reduction in falls 12mo post-session compared to 12mo pre-session (15% compared to 34% incidence).
Pai et al. [63]	3 groups tested +6, +9 & +12mo respectively: +6mo: *n* = 24, 13♀ 74.6 ± 5.8y; +9mo: *n* = 23, 15♀ 71.8 ± 5.5y; +12mo: *n* = 26, 19♀ 72.0 ± 4.7y. All community dwelling, healthy.	Slip (low friction moveable platform; slid up to 90 cm forward at foot contact).	24 slips in 37 gait trials.	Proactive & reactive stability (measured at touchdown of the to-be-perturbed step & the first recovery step respectively). % of falls following the lab perturbations.	Falls reduction from 42.5% to 0%. 0%, 8.7% & 11.5% of participants at the +6mo, +9mo & +12mo slips respectively fell. Stability improved & was better at all time points than the first slip.
Parijat and Lockhart [58]	Training: *n* = 12, 71.24 ± 6.82y. Control: *n* = 12, 74.18 ± 5.82y 12♂ 12♀. All community dwelling, healthy.	Pre-post: slippery surface. Training: slip (moveable platform; 30 cm at 1.2 m/s forward) with ±20% velocity based on ability.	12 slips in 24 gait trials.	Slip distance & peak sliding heel velocity pre- & post-training on the slippery surface. % of falls following the lab perturbations.	Falls reduction on slippery surface from 42% (pre) to 0% (post). The reduction in slip distance & peak sliding heel velocity was greater in the training group.
Sakai et al. [66]	*n* = 45, 26♀, 71.4 ± 3.6y. Community dwelling, healthy.	Treadmill decelerations at heel strike during walking at 2 km/h. 50% reduction in belt speed lasting 500 ms.	20 sudden treadmill belt decelerations at heel strike during 5mins of walking.	Peak forward & backward sacrum accelerations (accelerometer) within 1 gait cycle post-perturbation (average of first & last 10 perturbations).	Mean peak sacrum accelerations were lower in the final 10, compared to the first 10 perturbations.

♀: female; ♂: male; *mo* month, *MoS* margin of stability, *BoS* base of support, *X*_*CoM*_ extrapolated centre of mass

### Perturbation paradigms

In the nine included studies, moveable floor platforms used for simulating slips were the most commonly used perturbation type [55, 57, 58, 62, 63]. In these five studies, participants walked at self-selected speeds [55, 57, 58, 62, 63]. In four of these five studies, the platforms could freely slide up to 90 cm whereas in the other study, the velocity was controlled (limited to a velocity of 1.2 m/s and maximum acceleration of 20 m/s^2^) and the maximum displacement was only 30 cm [58]. In the 90 cm sliding platform condition, the platforms were unlocked at foot touchdown, detected by the force plates, using a mechanical locking mechanism. The papers describe the platforms as low friction and while exact velocity of the platforms’ slides are not reported (these will have varied due to different walking velocities and limb or body configurations at touchdown), the percentage of older adults who fell during the first slip ranged from 42.5 to 56%, indicating the reasonably high magnitude and impact of the perturbation [55, 57, 62, 63]. These four studies used a protocol containing 24 slips in 37 walking trials, whereas Parijat and Lockhart [58] applied 12 slips in 24 walking trials.

Ground surface compliance changes were applied in two of the included studies [64, 65]. This perturbation consisted of a section of the walkway that could be replaced with a soft element without a visible difference to the normal hard surface. The soft element was composed of a 17 cm thick piece of foam with an average deformation of about 10 cm for the participants [64, 65]. In both studies, the measurements began with three baseline walking trials where the hard element was used [64, 65]. In the first study, 19 walking trials were conducted after baseline, where only the 2nd, 8th and 19th trials used the hard surface [64]. While this paradigm was used to assess predominantly predictive, feedforward locomotor adaptation during repeated soft surface trials, we may assume that reactive, feedback-driven locomotor adaptations played a role in the first four soft surface trials, as the participants were not aware which surface would be used on a given trial. The second study was, however, specifically designed to assess reactive adaptation, where 28 trials in total were conducted, with only five soft surface trials interspersed throughout the hard surface trials [65]. Improvements in stability control were seen during both the first four soft surface trials in the first study [64] and by the fourth soft surface trial of the second study [65]. However, as these studies did not report numbers of lab or daily life falls, it is difficult to determine the impact such a perturbation paradigm could have, when used as training, on daily life falls incidence. In these studies, walking speed was set at 60% of walk-to-run velocity, based on walking trials conducted before the perturbation trials [64, 65].

Finally, two studies used treadmill belt accelerations or decelerations [56, 66] to perturb the gait of their participants. Sakai et al. [66] used a deceleration perturbation during walking at 2 km/h, resulting in a 50% reduction in belt speed at heel strike over 0.5 s. This perturbation was applied 20 times during 5 min of walking. Lurie et al. [56] used a combination of treadmill belt accelerations and decelerations during both stance and gait, but did not report the exact number of perturbations used in their study, as the perturbation type (stance or gait), magnitude (scale of 1–5; no velocity/acceleration values reported) and number was determined by a physical therapist for each participant individually. The authors determined the perturbation magnitude based on the treadmill belt pulse peak velocity, elapsed time to peak velocity, elapsed time during which the peak velocity was maintained, and time required to decelerate the treadmill belt to zero velocity, but these values or ranges were not reported [56]. On average, the participants completed approximately six sessions of 45 min and the sessions were progressive in terms of perturbation magnitude, based on the physical therapist’s judgement [56].

### Reactive recovery responses and falls reduction in the included studies

In the included studies, perturbation paradigms including four [64], five [65], 12 [58] and 24 [55, 57, 62, 63] perturbations led to improved reactive recovery responses to the disturbances, with one study showing transfer to another perturbation task [58]. Lurie et al. [56] reported that the mean perturbation magnitude of successfully negotiated perturbations significantly increased from the first to final session. Sakai et al. [66] found that the mean peak anteroposterior acceleration (determined using an accelerometer attached to the sacrum) was significantly reduced in the final ten perturbations, in comparison to the first ten, indicating an improved reactive recovery response. Four of the included studies reported a reduction in the percentage of participants who fell from 42.5 to 56% during the first perturbation, to 0% following 12 [58] and 24 [55, 62, 63] perturbations, with one of the 24 perturbation studies reporting a reduction to 5% after only five perturbations [62]. Pai et al. [57] reported a 50% reduction in daily life falls in the 12 months following the perturbation session. Lurie et al. [56] reported that their intervention group participants experienced fewer falls (19% vs. 33%) and fewer falls that led to injuries (8% vs. 18%) in comparison to the control group, but these were not statistically significant as the study was not powered to detect changes in falls incidence.

PEDro Scale scores of the included articles can be found in Table 2. The mean score of all articles was 3.33, with only three studies receiving a score of four or higher. The levels of evidence for the different perturbation paradigms of the included studies are presented in Table 3. Based on the definitions provided by Teasell et al. [61], strong evidence exists only for moveable platform perturbations, as two or more RCTs have demonstrated beneficial effects of experiencing this type of perturbation on reactive recovery responses and falls incidence. Moderate evidence exists for treadmill-based perturbations as only one RCT has reported beneficial effects to date. Finally, the level of evidence for surface change perturbations was limited, as no RCTs have been conducted using this type of gait perturbation.

**Table 2:** PEDro Scale scores for individual studies included in this review

Study	PEDro Scale Item											
	1^a^	2	3	4	5	6	7	8	9	10	11	Total
Bhatt et al. [55]^b^	Yes	1	0	1	0	0	0	0	0	1	1	4
Bierbaum et al. [64]	No	0	0	0	0	0	0	0	0	1	1	2
Bierbaum et al. [65]	No	0	0	0	0	0	0	0	0	1	1	2
Lurie et al. [56]^b^	Yes	1	1	1	0	0	0	1	0	1	1	6
Pai et al. [62]	Yes	0	0	0	0	0	0	0	0	1	1	2
Pai et al. [57]^b^	Yes	1	0	1	0	0	0	0	1	1	1	5
Pai et al. [63]	Yes	0	0	1	0	0	0	0	0	1	1	3
Parijat and Lockhart [58]	No	1	0	0	0	0	0	0	0	1	1	3
Sakai et al. [66]	No	0	0	0	0	0	0	1	1	0	1	3

PEDro Scale Items: 1: Eligibility criteria were specified; 2: Subjects were randomly allocated to groups; 3: Allocation was concealed; 4: The groups were similar at baseline regarding the most important prognostic indicators; 5: There was blinding of all subjects; 6: There was blinding of all therapists who administered the therapy; 7: There was blinding of all assessors who measured at least one key outcome; 8: Measures of at least one key outcome were obtained from more than 85% of the subjects initially allocated to groups; 9: All subjects for whom outcome measures were available received the treatment or control condition as allocated or, where this was not the case, data for at least one key outcome was analysed by “intention to treat”; 10: The results of between-group statistical comparisons are reported for at least one key outcome; 11: The study provides both point measures and measures of variability for at least one key outcome. Ratings: No/unclear = 0, Yes = 1

^a^ Not included in total score

^b^ Scores obtained from PEDro website (http://www.pedro.org.au)

**Table 3:** Level of evidence per perturbation type for improving reactive responses and/or falls risk

Perturbation Type	Studies Reporting Beneficial Effects		Negative Studies (with sufficient power)		Level of Evidence^a^
	RCTs	Non-RCTs	RCTs	Non-RCTs	
Moveable floor platform	3	2	0	0	Strong
Treadmill (acceleration/deceleration)	1	1	0	0	Moderate
Surface Change	0	2	0	0	Limited

^a^Level of evidence based on Teasell et al. [61]: Strong Evidence: Two or more RCTs with PEDro scores of 4 or higher; Moderate Evidence: One RCT with a PEDro score of 4 or higher; Limited Evidence: At least one non-RCT (i.e. prospective or retrospective controlled trials, single group studies etc.)

## Discussion

The aim of this systematic review was to determine the variety, characteristics and effectiveness of methods that have been used to date for improving reactive recovery responses during walking and reducing falls among healthy older adults. To achieve this, a systematic search for studies with healthy older adults that applied unexpected mechanical disturbances during walking and assessed changes in reactive recovery responses or falls incidence was conducted. After screening, nine articles met the inclusion criteria. Moveable floor platforms [55, 57, 58, 62, 63], ground surface compliance changes [64, 65] and treadmill belt accelerations or decelerations [56, 66] have been used to perturb the gait of older adults with the aim of stimulating adaptations in the reactive response. Eight of the nine studies reported improvement in the reactive recovery response [55, 56, 58, 62–66], four studies reported a reduction in laboratory falls [55, 58, 62, 63] and two studies reported a reduction in daily life falls [56, 57]. As well as the range of perturbation types, the magnitude and frequency of the perturbations varied between the studies.

Regarding the number and magnitude of the moveable platform perturbations, one study [58] applied 50% fewer perturbations with a smaller magnitude in comparison to the other studies utilizing platforms [55, 57, 62, 63]. Despite this difference in magnitude and number of perturbations, all studies found statistically significant improvements in various measures, including a reduction in the number of trials where participants required support from the safety harness (classed as falls). Combined with the fact that one of the studies reported that the majority of the improvements occurred within the first five perturbation trials [62], it appears that healthy older adults can benefit from experiencing only a few moveable platform perturbations. This could be important for future research and application in clinical settings, as this implies that the minimum effective dose of such perturbations could be very low. However, more research is needed to determine if such a low number of perturbations would also yield long term benefits, in addition to these acute benefits seen in the lab.

In order to fully understand and interpret the results of gait perturbation studies, it is important to consider how walking speed and perturbation magnitudes were controlled or scaled based on the participants included. Concerning the studies included in this review, the five studies using moveable floor platforms had participants walking at self-selected speeds [55, 57, 58, 62, 63]. Other than walking speed, no scaling or standardisation of the paradigm based on the participants was conducted, apart from an increase or decrease of 20% platform slip velocity in the study of Parijat and Lockhart [58], which was based on participants’ performance during the session. In the two ground surface compliance change perturbation studies, walking speed was set at 60% of walk-to-run velocity, based on walking trials conducted before the main measurements and the perturbation itself was not adjusted based on the participants [64, 65]. Concerning the two treadmill-based paradigms, Sakai et al. [66] used a set walking speed of 2 km/h for all participants with no changes in the perturbation, while Lurie et al. [56] used individualised walking speeds and perturbation intensities based on the abilities of the participants (values or ranges of speeds and magnitudes were not reported) [56]. Due to individual differences in locomotor capacities, using the same walking speed for all participants (as in: [66]) may lead to some being more challenged than others by the perturbations applied. This may lead to floor or ceiling effects in the adaptation to perturbations, which may be particularly problematic when comparing groups of different locomotor capacities [67]. In the same manner, using the same (or similar) perturbations for participants with different capacities (as in: ([55, 57, 62–66]) may lead to similar issues, as one individual or group may require relatively more substantial adaptation than others to maintain stability. When using a self-selected (as in: [55, 57, 62, 63]) or individually standardised (as in: [64, 65]) walking speed, faster walking speeds may make stability recovery more difficult following a perturbation, compared to slower speeds, due to a higher forward velocity, and therefore a reduced margin of stability in the forward direction [68]. How the interaction of walking speed and perturbation magnitude influences reactive recovery responses and adaptation to perturbations in different age and patient groups remains a question for future studies.

As well as the type, number and magnitude of perturbations, perturbation direction may be an important feature of such paradigms with regard to daily life falls reduction. In the studies included in this review, perturbations were mostly applied in anterior or posterior directions. This is noteworthy, as it is well documented that mediolateral stability declines with age [69–73] and is related to falls incidence in older adults [74–77]. Additionally, there is evidence to suggest that adaptations to perturbations in one plane of motion do not necessarily transfer and benefit stability control in other planes of motion [48, 78, 79]. Regarding the type and direction of perturbations used to stimulate adaptation in the reactive recovery responses, it has been previously suggested that, due to the diversity of perturbations that can occur in daily life, it may be more effective to train the mechanisms of stability recovery, rather than focus on specific perturbations [80, 81]. Such an approach (whereby multidirectional stepping and counter rotating mechanisms to maintain balance are exercised) has been shown to result in an improvement in stability recovery following lab-based perturbations [80–82]. However, no study has yet looked at the effects of such an intervention on daily life falls incidence in older adults. Furthermore, if particular gait perturbation paradigms, like those described in this review, would also result in an improvement in these mechanisms, there may not be any reason to suspect a less positive outcome on daily life falls.

The mean PEDro Scale score of the included studies was 3.33, with only three studies receiving higher scores of four, five and six (Table 2). However, it should be kept in mind that blinding of the participants and staff members conducting such experiments or training is difficult, meaning that points five and six of the PEDro scale will generally not be met by such studies. Regarding the levels of evidence determined for the perturbation paradigms of the studies included in this review, it is important to note the relatively low number of RCTs conducted thus far, especially with paradigms other than moveable floor platforms. Until more RCTs are conducted with varying perturbation paradigms, concrete conclusions regarding the beneficial effects of different perturbations are difficult to make. Despite the strong evidence for moveable platform perturbations, based on the definitions provided by Teasell et al. [61], a number of advantages of treadmill setups should be highlighted. The first relates to the predictability of the perturbations applied in a gait lab setup with overground walking, in comparison to treadmill walking. In overground setups, the location of the perturbation on the walkway is usually constant, which means that even if the perturbation is not applied in every trial, the participant may make predictive, feedforward adaptations in their gait after identifying the location of the potential perturbation, facilitating better performance and adaptation. Shapiro and Melzer [83] have previously highlighted similar issues related to the same perturbation direction being used for all trials. In contrast, treadmill setups do not face this location issue, as accelerations or decelerations can be applied at any time during continuous walking, making it more difficult for participants to anticipate perturbations. As well as this, depending on the setup, perturbations in multiple directions could be used. Furthermore, treadmill perturbation setups may be more feasible in clinical settings [56] due to the smaller space required, in comparison to a gait lab and walkway. However, the type and magnitude of perturbations that can be applied may be limited by the size and capabilities of the treadmill.

While this systematic review found three main types of perturbations that have been used to examine and stimulate the reactive adaptation of gait in older adults, there are a number of other gait perturbation paradigms reported in the literature. These were excluded from this review due to the participant population used (i.e. not older adults), or that reactive adaptation was not analysed in the studies. Cable trip systems, in conjunction with either treadmill or overground walking have been used effectively on multiple occasions to analyse gait stability and adaptation in multiple participant groups [21, 31, 43, 49, 84]. Another method for initiating tripping responses is the use of objects popping up from the ground, inhibiting the swing phase of gait [85, 86]. As well as tripping methods, a few different methods have been used to trigger slipping responses in addition to the moveable platforms from the included studies described above. The slip perturbation used to test the participants in the study of Parijat and Lockhart [58] could also be used in a repetitive manner to stimulate adaptation. Additionally, different levels of shoe/floor friction using different materials have been used [87]. As well as trips and slips, a number of various waist push and pull methods have been applied during gait to analyse mediolateral stability in particular [78, 88, 89]. Finally, perturbations involving sudden surface height changes [90–92] or multiple changes in surface tilt, height and position [93] have been employed. The majority of these perturbations have been used to investigate some specific characteristic of gait stability or adaptability but few have been used for the purpose of training. Therefore, further research is needed before recommending these perturbations for training purposes among older adults.

One potential limitation of this review (and the studies included) is that it is difficult to determine if the responses to the perturbations were fully reactive in nature. In most movements, an interplay exists between reactive and predictive adaptations [50]. In order to reduce the influence of predictive adaptations, two steps can be taken. Firstly, the degree of predictability of the perturbations must be kept to a minimum (e.g. using catch trials, random timing etc.), and secondly, attempts can be made to assess pre-perturbation movement to assess if predictive adjustments are being made. As described above, the setup may also affect the predictability of the perturbations and results should be interpreted with this in mind. One study that we know of [94] has applied a truly unexpected perturbation, albeit with young participants, where participants were under the impression that they were taking part in a normal gait analysis and were subsequently perturbed. The effects of this perturbation were markedly greater compared to the more common situation where the participants knew that they would be perturbed at some point during the trial [94]. However, such a procedure has not been conducted with older adults.

It is important to note that in this review, we focussed on gait, as opposed to stance perturbation paradigms. As detailed in the introduction, perturbations applied during gait are theoretically more task specific to daily life falls among healthy older adults than stance perturbations, as most falls occur during walking in this population [5–9]. Additionally, forward velocity during gait may make stability recovery following a perturbation more difficult, as an increased walking speed, and thereby higher forward centre of mass velocity, results in a lower anterior margin of stability [68]. However, a decrease in falls incidence has been shown after four sessions of perturbations to stance in healthy older adults [95], indicating that stance perturbations may also be beneficial. To our knowledge, a direct comparison of the effectiveness of stance versus gait perturbations for falls reduction among older adults has not been made, and it is not known how repetition of either gait or stance perturbations would benefit performance of the other. Stance perturbations have often been applied in patient populations (for example: [96–98]), possibly due to practical reasons (simpler setup, easier quantification of stability) or perhaps due to the relatively lower demand of the tasks, in comparison to gait perturbations. This suggests that a progression could be made from stance to gait perturbations in clinical settings.

## Conclusions

To date, a range of perturbation paradigms (moveable floor platforms, ground surface compliance changes and treadmill belt accelerations or decelerations) have been used to perturb older adults while walking. As well as the range of perturbation types that have been applied, there is huge variation among studies in the number and magnitude of the perturbations. The fact that the majority of studies report improvements in participants’ ability to maintain stability following exposure to the perturbation paradigms is in one sense promising, as benefits appear to be produced from many different paradigms, but this restricts our understanding of the underlying mechanisms of improvement and what components of the paradigms are responsible for the improvements. The effects of perturbation type, magnitude and number on the extent of adaptation of the reactive recovery responses and the retention of such adaptations over longer time periods should be investigated in future research. This may lead to more efficient and effective perturbation paradigms and to information regarding the minimum effective dose for falls incidence reduction among healthy older adults.

## Additional file

Additional file 1: Search terms in PubMed, Web of Science, MEDLINE and CINAHL. (PDF 84 kb)

## Abbreviations

PEDro: Physiotherapy evidence database
RCT: Randomised controlled trial
