# Supplementary material for: A systematic review of gait perturbation paradigms for improving reactive stepping responses and falls risk among healthy older adults
Source: Eur Rev Aging Phys Act. 2017 Mar 2;14:3. doi: 10.1186/s11556-017-0173-7 (PMC5335723; doi:10.1186/s11556-017-0173-7)
Supplement: Additional file 1: — Search terms in PubMed, Web of Science, MEDLINE and CINAHL. (PDF 84 kb) [file 11556_2017_173_MOESM1_ESM.pdf]

Additional file 1 for: A systematic review of gait perturbation paradigms for improving reactive stepping responses and falls risk among healthy older adults

Additional file 1: Search terms in PubMed, Web of Science, MEDLINE and CINAHL

PubMed Search on 16/12/2015:
((((gait OR walking OR walk OR locomotion)) AND (Perturb* OR trip OR tripping OR slip* OR surface translation OR balance loss OR agility OR dynamic balance OR dynamic stability OR waist pull)) AND (training OR exercise OR adaptation OR adaptive OR repeated OR repetition OR rehabilitation OR task OR responses OR adjustments)) AND (Age OR ageing OR aging OR aged OR elderly OR old OR older OR senior)
Search returned: 1911 results

Web of Science on 16/12/2015:
(TS=(gait OR walking OR walk OR locomotion) AND TS=(Perturb* OR trip OR tripping OR slip* OR surface translation OR balance loss OR agility OR dynamic balance OR dynamic stability OR waist pull) AND TS=(training OR exercise OR adaptation OR adaptive OR repeated OR repetition OR rehabilitation OR task OR responses OR adjustments) AND TS=(Age OR ageing OR aging OR aged OR elderly OR old OR older OR senior)) OR (TI=(gait OR walking OR walk OR locomotion) AND TI=(Perturb* OR trip OR tripping OR slip* OR surface translation OR balance loss OR agility OR dynamic balance OR dynamic stability OR waist pull) AND TI=(training OR exercise OR adaptation OR adaptive OR repeated OR repetition OR rehabilitation OR task OR responses OR adjustments) AND TI=(Age OR ageing OR aging OR aged OR elderly OR old OR older OR senior))
Search returned: 1454 results

MEDLINE on 16/12/2015:
((gait or walking or walk or locomotion) and (Perturb* or trip or tripping or slip* or surface translation or balance loss or agility or dynamic balance or dynamic stability or waist pull) and (training or exercise or adaptation or adaptive or repeated or repetition or rehabilitation or task or responses or adjustments) and (Age or ageing or aging or aged or elderly or old or older or senior)).mp. [mp=title, abstract, original title, name of substance word, subject heading word, keyword heading word, protocol supplementary concept word, rare disease supplementary concept word, unique identifier]
Search returned: 970 results

CINAHL on 16/12/2015:
TX (gait OR walking OR walk OR locomotion) AND TX (Perturb* OR trip OR tripping OR slip* OR surface translation OR balance loss OR agility OR dynamic balance OR dynamic stability OR waist pull) AND TX (training OR exercise OR adaptation OR adaptive OR repeated OR repetition OR rehabilitation OR task OR responses OR adjustments) AND TX (Age OR ageing OR aging OR aged OR elderly OR old OR older OR senior)
Search returned: 888 results

In total: 5223 records
After removal of duplicates: 3332
After title screening: 219
After abstract screening: 27
After full text screening: 9
